# A randomized controlled trial of resistance and balance exercise for sarcopenic patients aged 80–99 years

**DOI:** 10.1038/s41598-020-75872-2

**Published:** 2020-10-30

**Authors:** Yuxiang Liang, Renjie Wang, Jiaojiao Jiang, Lingling Tan, Ming Yang

**Affiliations:** 1 Rehabilitation Medicine Center, West China Hospital, Sichuan University, No. 37 Guoxue Lane, Chengdu, China; 2 Center of Gerontology and Geriatrics, West China Hospital, Sichuan University, No. 37 Guoxue Lane, Chengdu, China; 3 Precision Medicine Research Center, West China Hospital, Sichuan University, No. 37 Guoxue Lane, Chengdu, China

**Keywords:** Health care, Medical research, Clinical trial design, Clinical trials, Randomized controlled trials

## Abstract

We compared a mixed exercise program (i.e., balance exercise plus resistance exercise) with resistance exercise in a single-blind, randomized controlled trial in a post-acute care unit. In total, 60 sarcopenic patients were randomly assigned to an intervention group (12-week mixed exercise) and a control group (12-week resistance exercise). The primary outcomes were the change of the Barthel Index and the number of fallers. The intervention group showed a mean increase of 9.5 points on the Barthel Index (95% confidence interval (CI) 3.9–15.1), while the control group showed a mean increase of 6.3 points (95% CI 2.3–10.4). The mixed exercise program provided a significant benefit over resistance exercise (adjusted mean difference of the change of Barthel Index: 6.8 points; 95% CI 1.4–12.1). The number of fallers was 13.3% and 23.3% in the intervention and control groups, respectively, but the difference was not significant (risk ratio (RR) 0.89, 95% CI 0.69–1.13, *p* = 0.506). In conclusion, compared with resistance exercise, the mixed exercise program appears to further improve the activities of daily living and physical performance in our study population. Under the monitoring of experienced physiotherapists, both exercise programs are feasible and safe for this population.

## Introduction

Sarcopenia is a disease defined by loss of muscle mass and muscle function^1^. It has been proven to be associated with numerous adverse health outcomes, such as functional impairment, falls, poor quality of life, and even death^1,2^. The prevalence of sarcopenia varies significantly in different populations due to the lack of unique diagnostic criteria and different clinical features of study populations^3^. However, there is a consensus that the prevalence of sarcopenia increases with advancing age^1,2,4^. Meanwhile, older adults in acute care settings or post-acute care settings are more prone to sarcopenia than their community-dwelling counterparts^1,4,5^.

At present, the only candidate with robust evidence for treating sarcopenia in older adults is exercise^6–8^. Unfortunately, the best approach to exercise sarcopenic patients has not yet been established^9,10^. Resistance exercise is the most commonly suggested exercise regimen for sarcopenia by recent consensuses^2,4^. However, a recent overview of systematic reviews indicated that the majority of exercise interventions for sarcopenia were mixed exercise programs including aerobic, balance, and resistance exercise (instead of resistance exercise alone)^11^. Whether mixed exercise programs are better than resistance exercise alone for sarcopenic patients has not been reported.

Additionally, with an aging population has become the most rapidly increasing population segment in many countries^7^. Old people are generally frailer and sicker than their younger counterparts^7^; therefore, it is important to research the effectiveness, feasibility, and safety of exercise for older populations. At present, there is limited evidence regarding best-practice exercise for old people in post-acute settings.

Post-acute care refers to medical services that support patients’ recovery from acute illness or management of chronic conditions or functional disabilities. Older patients in post-acute care settings differ considerably from community-dwelling older adults with regard to their physical performances and comorbidities^12^. A recent systematic review demonstrated that exercise interventions are effective for preventing falls in community-dwelling older adults, but they might not have the same efficacy when transferred to post-acute care settings^13^. Therefore, the recommendations regarding exercise interventions for sarcopenic older patients in post-acute care settings should not be established only based on studies conducted in community-dwelling older adults. We systematically searched PubMed and Embase databases to identify four studies that addressed exercise programs for sarcopenic patients in post-acute care settings^14–17^. Of them, three were conducted in nursing homes^14–16^, while the other was conducted in a subacute care unit^17^. However, most of these studies had no control group and none was designed as a randomized controlled trial (RCT).

To address this important clinical question, we conducted an RCT to compare the effectiveness and feasibility of a mixed exercise program (i.e., balance exercise plus resistance exercise) with resistance exercise for treating sarcopenia in older patients in a post-acute care unit.

## Methods

### Study design

We conducted a single-center, two-arm, parallel-group and single-blind RCT according to the CONSORT statement of transparent reporting^18^ and SPIRIT 2013^19^. We conducted this RCT in the post-acute care unit of the center of gerontology and geriatrics in a tertiary public hospital in Chengdu, China.

The study protocol was registered in ClinicalTrials.gov (ID: NCT04216368, the date of registration: 02/01/2020). The study protocol was approved by our hospital’s Biomedical Ethics Committee. We conducted the study in accordance with the ethical standards set by the 1964 Declaration of Helsinki and its later amendments. All participants (or their legal representatives) signed written informed consent.

### Participants and randomization

In this study, we focused on older patients with sarcopenia, as well as stable comorbidities with a level of functional reserve and cognitive capacity high enough to allow them to follow a physiotherapist and perform exercise programs. Therefore, a physiotherapist (R.W.) conducted a screening interview within the first 48 h of admission from acute care units in our hospital to determine eligible patients using the following inclusion criteria: aged 80 years or older with sarcopenia defined by the recommendation from the Asian Working Group for Sarcopenia (AWGS)^20^; ambulate capabilities (assistance was allowed if necessary); and ability to communicate and collaborate with medical staff. The exclusion criteria were as follows: terminal illness, acute lower respiratory infection, uncontrolled arrhythmias, uncontrolled heart failure, recent myocardial infarction, uncontrolled respiratory failure, acute pulmonary embolism, recent major surgery, recent dialysis, a bone fracture in the past 3 months, or expected length of stay less than 12 weeks.

All the included patients received a baseline assessment, including history of falls in the past year and current nutrition status evaluated using the mini nutritional assessment (MNA)^21^. The total score of the MNA ranged from 0 (worst) to 30 (best) points. An MNA score of < 17 points indicated malnutrition, while a score between 17 and 23.5 points indicated “malnutrition risk”^21^. We also estimated the appendicular skeletal muscle mass (ASM) of each patient using a bioimpedance analysis (BIA) device (InBody 230, Biospace Co.Ltd., Korea). The appendicular skeletal muscle mass index (ASMI) was then calculated with the equation: ASMI (kg/m^2^) = ASM/ height^2^^20^. In addition, the following data were collected from the local hospital information system: age, sex, education level, body mass index (BMI), and comorbidities (i.e., diabetes, hypertension, stroke, chronic obstructive pulmonary disease, and coronary heart disease). Moreover, the outcomes described below were also evaluated at baseline.

After the baseline assessment was performed, participants were randomly assigned with a 1:1 ratio into either the intervention group or control group. The random assignment was performed using a Researcher Randomizer. The outcome assessment staff and statistical analyzer were blind to the study design and group allocation.

### Intervention

Before the start of intervention, participants in both groups and their caregivers were educated and familiarized with the training procedures. An experienced physiotherapist (Y. L.) provided instructions and encouragement, supervising each patient session during their exercise. In both groups, the exercise interventions were individually designed with twice-weekly sessions for 12 weeks and closely supervised by a physiotherapist.

The intervention group received a mixed exercise program including balance and resistance exercise. In each session, the participants received a light 5-min warm-up followed by 20 min of targeted balance training. Next, they were allotted a five-minute rest before another 20 min of resistance training. Each session ended with a 5-min cool-down that incorporated stretching. To be specific, the balance exercise program included: heel and toe raise and static balance in weeks 1–3; varied directional quick stepping in weeks 4–6; reaching and single-leg standing in weeks 7–9; heel to toe walking and complex cross-over stepping activities in weeks 10–12. The resistance exercise included leg press, leg extension and flexion, leg abduction and adduction, chest press, and seated row. The individual loads of resistance training were determined based on the strength test at the first intervention and at the 13th session. Resistance exercise was performed at 70–80% of one-repetition maximum, 3 sets of 8–12 repetitions each (with a 2-min rest between sets).

The control group engaged in a resistance exercise program. In each session, participants received a light 5-min warm-up first followed by 20 min of resistance training. Next, they were allotted a five-minute rest before another 20 min of resistance training. Each session also ended with a 5-min cool-down. The resistance exercise was the same as the intervention group.

Furthermore, patients in both groups received standard care for their chronic comorbidities by the geriatricians.

### Outcomes

The primary outcomes were the improvement of activities of daily living (ADL) and the number of fallers during the 12-week period. The improvement of ADL was evaluated by the change in the Barthel Index from baseline to the end of the interventions. The Barthel Index is a 10-item scale of 0 (worst) to 100 (best) points, which has been widely used to evaluate ADL in both research and clinical practice^22^. The clinically significant change was 5 points for the Barthel Index score^23^.

The secondary outcomes were as follows. First, the change of the short physical performance battery (SPPB) score, which is a valid tool for assessing lower extremity function. The total score of SPPB ranged from 0 (worst) to 12 (best) points^24^. Second, the change of a 4-m usual gait speed was scored. Third, the change of handgrip strength was measured using a digital grip dynamometer (EH101, Xiangshan Inc., Guangdong, China) to the nearest of 0.1 kg. The recommendations from the Chinese National Physical Fitness Evaluation Standard were used as the handgrip strength measurement protocol^25^. Fourth, the change of Berg balance score was scored, which is a valid tool for assessing balance function in clinical practice^26^. The score of Berg balance ranged from 0 (worst) to 56 (best) points. Fifth, the change of the timed “UP and GO” test (TUG) score was used, which is a widely used tool for assessing functional mobility^27^. The patients were asked to rise from an armchair, walk three meters at a comfortable pace, turn, walk back, and sit down again^27^. The time to complete the whole process was recorded to the nearest of 0.1 s. Lower timed scores indicate better functional mobility. Sixth, any adverse event related to the interventions during the study period were noted, including falls, pain, injury, bone fracture, cerebrovascular accident, stroke, and the transfer to the acute unit.

### Statistical analysis

To achieve 80% power at an α level of 0.05 (2-sided), we needed 25 patients in each group to detect a mean difference in change for the Barthel Index of 5 points during the 12-week period. This was based on a previous study that showed a significant improvement of the Barthel Index (20.4 ± 18.3 points) after 3 months of physical rehabilitation among sarcopenic older patients^17^. Based on our previous experience; we estimated an attrition rate of 10%. Therefore, at least 55 patients were required to ensure that there were sufficient participants at the end of the intervention.

We used the intention-to-treat approach for all analyses. Missing data were imputed by using “the conservative assumption that patients with missing data in the intervention group had the same rate of falls as observed in the control group and vice versa”^28^ (for the outcome of fallers) and by setting the change across time to be zero (for the other outcomes)^29^. The normality of the distribution of continuous data was evaluated using the Kolmogorov–Smirnoff test. Group differences at baseline were compared using the χ^2^ test for categorical data and the ANOVA for continuous data. Group differences for all outcomes (except for the number of fallers) were analyzed using analyses of covariance^30^. The change of these outcomes from the baseline to the end of the interventions were the dependent variables in these analyses, while the group assignment was the independent variables. We adjusted these analyses for age, sex, baseline scores, education level, comorbidities, and nutrition status. The group difference in the number of fallers was compared using the χ^2^ test.

All statistical analyses were performed in SPSS version 25 (IBM Corp., Armonk, NY, US) and R software, version 3.5.3 (R Foundation for Statistical Computing, Vienna, Austria). All comparisons were 2-sided with an α level of 0.05 indicating statistical significance.

## Results

### Trial participants

**Figure 1:**
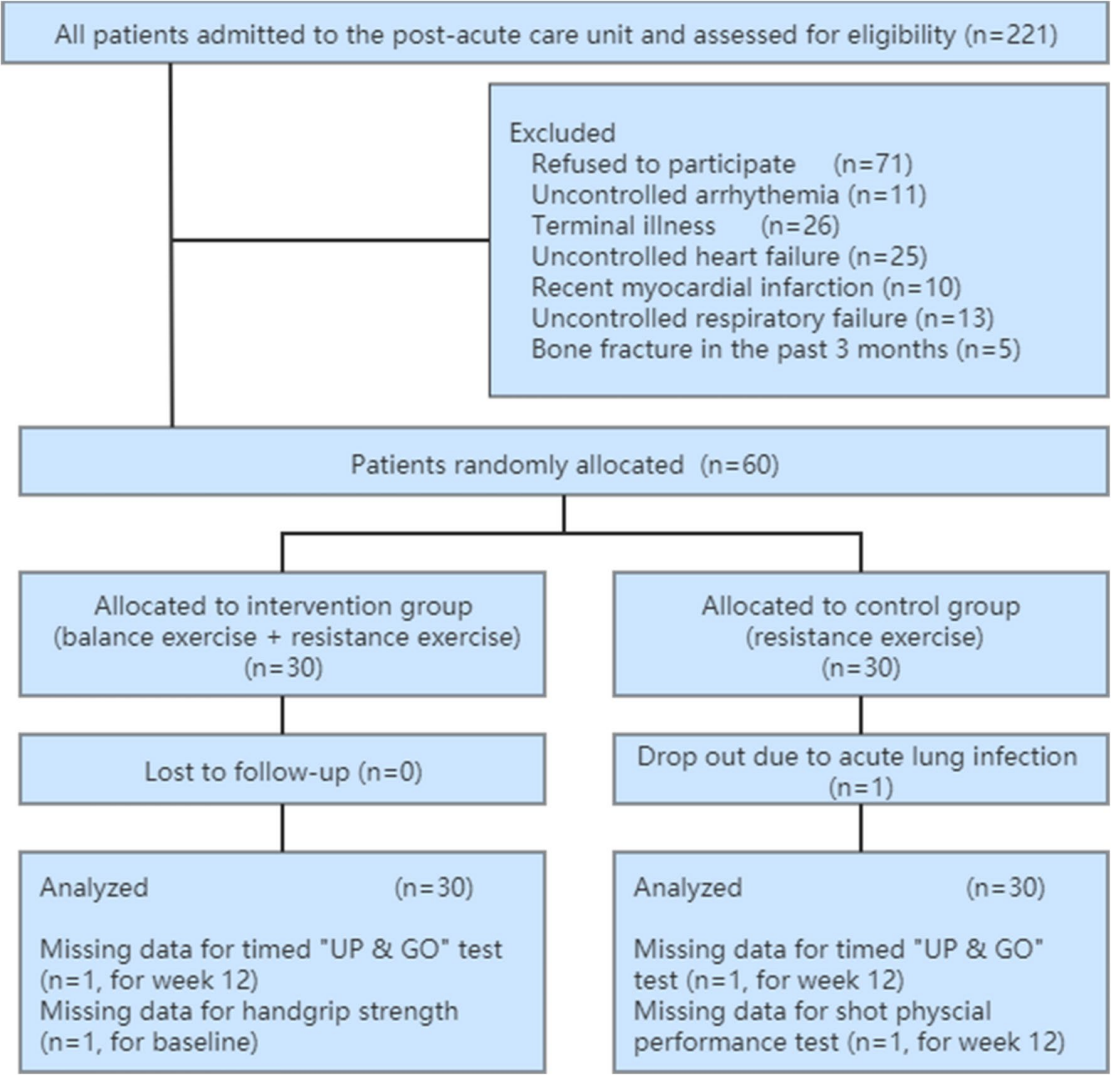
Flowchart of enrolment and randomization. An intention-to-treat approach was performed.

Figure 1 shows the study flow diagram. From June 2017 to January 2019, a total of 221 patients were screened, of whom 60 were included in the trial and underwent randomization. Next, 30 participants were assigned to the intervention group (receiving a mixed exercise program including balance and resistance exercise) and 30 to the control group (receiving a resistance exercise program). The participants in both groups also received standard care. One participant in the control group dropped out at the 13 weeks of intervention due to an acute lung infection. All remaining participants finished the 24 exercise sessions.

Patients characteristics at baseline are presented in Table 1. The mean age of the study cohort was 87.3 ± 5.4 years (range, 80–99 years, with 24 patients (40%) were nonagenarians). In total, 26 (43.3%) patients were women. There were no significant differences between groups at baseline for demographic and medical characteristics or for the study outcomes except for the TUG score, which was higher in the control group.

**Table 1:** Baseline characteristics of the study population.

	Intervention n = 30	Control N = 30	*p*
Age (years)	87.3 (6.0)	86.8 (4.7)	0.490
Women (%)	15 (50.0)	11 (36.7)	0.297
BMI (kg/m^2^)	21.6 (2.1)	21.0 (2.7)	0.367
*Education level (%)*			
≤ 6 years	9 (30.0)	9 (30.0)	0.301
7–12 years	14 (46.7)	9 (30.0)	
≥ 13 years	7 (23.3)	12 (40.0)	
*Comorbidities (%)*			
Diabetes	4 (13.3)	8 (26.7)	0.197
Hypertension	18 (60.0)	15 (50.0)	0.436
Stroke	7 (23.3)	5 (16.7)	0.519
Chronic obstructive pulmonary disease	8 (26.7)	6 (20.0)	0.542
Coronary heart disease	4 (13.3)	10 (33.3)	0.067
Patients with at least a fall in the past year (%)	9 (30.0)	10 (33.3)	0.781
*Nutrition status (%)*			
Malnutrition	13 (43.3)	17 (56.7)	0.579
Malnutrition risk	14 (46.7)	11 (36.7)	
Barthel index score	72.3 (17.3)	64.5 (15.4)	0.069
ASMI	5.9 (1.4)	6.2 (1.1)	0.179
Gait speed (m/s)	0.43 (0.15)	0.35 (0.17)	0.063
Handgrip strength (kg)	16.3 (4.8)	14.9 (6.1)	0.328
SPPB score	4.3 (1.4)	3.8 (1.8)	0.215
Berg balance score	20.8 (15.1)	17.7 (10.2)	0.345
TUG score	23.4 (11.2)	40.4 (42.4)	0.042

*ASMI* appendicular skeletal muscle mass index, *SPPB* short physical performance test, *TUG* timed up to go test.

Patients in the intervention group received mixed exercise program (balance exercise plus resistance exercise); while patients in the control group received resistance exercise alone. Patients in both groups also received standard care. Data are presented as mean (standard deviation) or n (percentage).

### Primary outcome: improvement of ADL

At the end of the interventions, the mixed exercise group showed a mean increase of 9.5 points on the Barthel Index (95% CI 3.9–15.1 points), while the resistance exercise group showed a mean increase of 6.3 points (95% CI 2.3–10.4 points). The mixed exercise program provided a significant benefit over the resistance exercise program (adjusted mean difference of the change of Barthel Index: 6.8 points, 95% CI 1.4–12.1 points, Table 2).

**Table 2:** Comparison of the primary and secondary outcomes by groups.

Outcomes	After 12 weeks				Change from the baseline				Adjusted group difference in mean change over 12 weeks^†^		F^†^	*P*^†^
	Control* n = 30		Intervention* n = 30		Control* n = 30		Intervention* n = 30					
	Mean	95% CI	Mean	95% CI	Mean	95% CI	Mean	95% CI	Mean	95% CI		
*Primary outcome*												
Barthel Index score	70.8	66.1–72.7	81.8	78.4–85.3	6.3	2.3–10.4	9.5	3.9–15.1	6.8	1.4–12.1	6.440	0.014
*Secondary outcomes*												
Gait speed (m/s)	0.40	0.35–0.46	0.54	0.49–0.61	0.05	0.02–0.07	0.12	0.06–0.18	0.07	0.01–0.14	4.347	0.042
Handgrip strength (kg)	16.3	14.0–18.7	20.5	18.1–23.0	1.5	0.8–2.2	4.3	2.1–6.4	3.3	0.7–5.9	6.626	0.013
SPPB score	4.6	4.0–5.2	6.6	5.7–7.5	0.8	0.4–1.2	2.3	1.3–3.3	1.6	0.5–2.8	8.396	0.006
Berg balance score	22.0	18.1–25.9	31.3	26.0–36.5	4.3	2.0–6.6	10.4	4.9–16.0	6.7	− 0.4–14.0	3.649	0.062
TUG score	31.7	25.4–37.9	18.5	16.1–20.9	− 8.5	− 22.1–5.2	− 4.7	− 8.7–− 0.8	2.0	− 14.8–18.8	0.056	0.813
MRMI score	29.4	27.2–31.7	34.6	33.0–36.2	0.8	− 0.2–1.8	4.2	1.9–6.5	3.1	0.6–5.7	6.310	0.015

*CI* confidential interval, *MRMI* modified Rivermead Mobility Index, *SPPB* short physical performance test, *TUG* timed up to go test.

*Patients in the intervention group received balance and resistance training; while patients in the control group received resistance training. Patients in both groups also received personalized oral nutrition supplements and standard care.

^†^Between group change by analysis of covariates (adjusted for baseline, age, sex, education level, comorbidities, and nutrition status).

### Primary outcome: number of fallers

During the 12-week period, the number of fallers was 13.3% (4/30) in the intervention group and 23.3% (7/30) in the control group, but the difference between groups was not statistically significant (risk ratio (RR) 0.89, 95% CI 0.69–1.13, *p* = 0.506).

### Secondary outcomes

Both the mixed exercise program and resistance exercise program improved gait speed, handgrip strength, SPPB score, and Berg balance score, but not the TUG score (Table 2). Compared with resistance exercise, the mixed exercise program significantly improved usual gait speed, handgrip strength, and the SPPB score, but not the Berg balance score and TUG score (Table 2).

In addition, no advance events related to the interventions were recorded during the study process.

## Discussion

To our knowledge, our study is the first RCT to compare a mixed exercise program with a resistance exercise program among older patients with sarcopenia in post-acute care settings.

Although the key role of resistance exercise for treating sarcopenia has been established^7^, the current evidence is mainly based on clinical trials conducted in community-dwelling older adults^31^. After systematically searching PubMed and Embase, we failed to identify any previous RCT to address this issue in post-acute care settings. However, two longitudinal intervention studies (without control arms) indicated that resistance exercise might be effective for improving muscle strength and physical performance in older adults living in post-acute care settings^14,15^. Chiu et al.^14^ observed that 12-week resistance exercise improved handgrip strength and functional independence in 64 older adults with sarcopenic obesity in long-term care facilities. Moreover, del Campo Cervantes et al.^15^ reported that the 12-week resistance exercise program improved handgrip strength and physical performance in 19 sarcopenic older adults living in a nursing home. Our study provides new evidence to support resistance exercise to be used in older adults living in post-acute care settings.

Our study found that both resistance exercise and mixed exercise programs improve ADL in older sarcopenic patients in post-acute care units, but the latter is better than the former. This finding supports the recent recommendation on exercise for older adults living in long-term care facilities: “The best exercise type is multicomponent training. . .other exercise types, particularly flexibility and balance, should be added to the exercise program whenever possible”^32^. This recommendation, however, was not especially designed for sarcopenic patients. No previous study directly compared the effect of two exercise programs on sarcopenia in this special population. However, a recent prospective study found that the rehabilitation program (including mobility training and endurance training) was effective for improving ADL in older sarcopenic patients in a subacute care unit^17^. In addition, another study showed that resistance and balance exercise improved muscle strength and reduced body fat in 42 very old residents living in nursing homes^16^.

Our study showed that the number of fallers was not significantly different between groups, although it appeared to be lower in the mixed exercise group than in the resistance exercise group. One possible reason is that the sample size of our study was too small to achieve the statistical power to detect differences between the two groups. In addition, we only assessed the number of fallers within 12 weeks. The effects of the two exercise programs for preventing falls in a longer period needs to be addressed in the future.

Other major concerns of exercise for older adults who commonly have multiple comorbidities and functional decline are feasibility and safety. We found no adverse events related to the exercise programs during the study period. Moreover, most of our participants completed all exercise sessions except for one who dropped out due to an acute lung infection. There is growing evidence to support the feasibility and safety of well-designed exercise programs for older adults. For example, a recent RCT indicated that resistance, balance, and walking exercises (2 daily sessions) was safe and effective to reverse the functional decline in older inpatients during acute hospitalization^33^. In addition, Hassan et al.^16^ reported that resistance and balance exercise programs were safe and feasible for older adults (approximately 1/3 having sarcopenia) living in nursing homes. On the other hand, based on our experience and findings from the literature^1,2^, older patients generally have difficulty or lack of motivation to participate in exercise programs. Therefore, encouragement and monitoring during exercise appears to be essential, especially for those in functional decline.

Our study has some limitations. First, we only assessed the post-intervention effects of the two exercise programs; however, the long-term outcomes are supposed to be more important in older patients with sarcopenia. According to a recent overview of systematic reviews, this was a common limitation shared across the relevant literature^11^. Therefore, the long-term effectiveness of different exercise programs needs to be evaluated in the future. Second, because our participants were very old, a physiotherapist in our team monitored the whole process of exercise to ensure the patients’ safety. Thus, these exercise programs are time-consuming and labor-intensive. As a result, the feasibility of transferring these interventions in real-world post-acute care settings may be compromised due to the lack of adequate physiotherapists or relevant medical staff. An individual exercise program may not be as cost-effective as a group-based exercise program, which has been proven to be effective for improving muscle function in institutionalized older adults^34,35^. Further studies are required to compare the two types of exercise for older sarcopenic patients in long-term care settings. Third, we did not assess some important outcomes, such as the change of lower limb muscle strength and muscle mass. Therefore, we could not provide a possible explanation for improved ADL and its relation to the interventions. Fourth, the follow-up duration of our study was relatively short and the sample size was small. Fifth, we performed ITT analyses and imputed missing data using previous reported methods^28,29^. These methods might have introduced bias into our results. However, as shown in Fig. 1, there were only four cases with a few missing data for the three variables. Therefore, our results are not significantly influenced. Lastly, although we randomly allocated the participants into two groups, their clinical characteristics were not fully balanced between the groups at the baseline, which would be expected from a simple randomization protocol with less than 200 cases. For example, the TUG score was significantly lower in the intervention group than in the controlled group (*p* = 0.042). Thus, our results might have been influenced by possible selection bias.

## Conclusions

Compared with resistance exercise, the mixed exercise program (balance exercise plus resistance exercise) appeared to have improved the activities of daily living, strength, and physical performance among older sarcopenic patients in post-acute care settings. Thus, we conclude that both exercise programs are feasible and safe for this population. Based on our experience, the exercise programs should be personally modulated and performed under the guide and monitoring of experienced physiotherapists.

Some essential questions regarding exercise interventions for older sarcopenic patients are still pending. For example, a recent systematic review of 25 RCTs revealed a dose–response relationship of resistance training to improve muscle strength and morphology in healthy old adults^36^; however, the optimal dose (e.g., intensity, volume, rest, frequency, repetition) and type (e.g., aerobic, balance, resistance, Tai Chi) of exercise for treating or preventing sarcopenia remains unclear. Large, multicenter, and well-designed RCTs are therefore warranted to address these issues in different clinical settings.

## Data Availability

The datasets generated during and/or analyzed during the current study are available from the corresponding author on reasonable request.
